# A recurrent sialolipoma of the parotid gland: A case report

**DOI:** 10.3892/ol.2014.2026

**Published:** 2014-04-02

**Authors:** PO-HAN LEE, JIANN-JY CHEN, YUNG-AN TSOU

**Affiliations:** 1 Department of Otolaryngology, Taipei City Hospital, Taipei 10629, Taiwan, R.O.C.; 2 Department of Otolaryngology Head and Neck Surgery, China Medical University, Taichung 40447, Taiwan, R.O.C.; 3 Department of ^3^ Otolaryngology, China Medical University Hospital, Taichung 40447, Taiwan, R.O.C.; 4 Department of Neurology, China Medical University Hospital, Taichung 40447, Taiwan, R.O.C.

**Keywords:** recurrent sialolipoma, parotid gland, parotidectomy

## Abstract

Sialolipoma is a rare benign neoplasm and was recently described as a histological variant of intraoral lipoma. In the current report, the case of a of a 65-year-old female with a slow-growing mass in the right parotid gland with recurrence is presented. The initial clinical diagnosis was a benign salivary gland tumor. The tumor was situated between the right parotid gland and the right masseter muscle; therefore, a superficial parotidectomy was performed. Histopathology revealed that the tumor was a sialolipoma of the parotid gland. During the three-month follow-up, a recurrent right parotid tumor was identified in the deep lobe space of the right parotid gland and a deep lobe parotidectomy was performed. The present case demonstrates that although surgical excision is generally sufficient to treat parotid gland sialolipoma, postoperative follow-up is required as multifocal lesions may potentially remain, which could result in recurrence.

## Introduction

Sialolipoma is a rare histological variant of intraoral lipoma, characterized by the well-circumscribed proliferation of mature adipose tissue and entrapped normal salivary glandular elements (1). Currently, there are >30 cases of sialolipoma reported in the literature. The overall incidence of salivary gland tumors in adults is 0.3% (1). Sialolipoma is prevalent in the parotid gland (2), with ~90% of cases occurring in adults (3); however, cases in infants have been reported (4). Previous studies identified no local recurrence, malignant transformation or other complications following conservative surgical excision (2–3,5). However, the present report demonstrates an exceptional case of parotid gland sialolipoma with recurrence following the performance of a superficial parotidectomy, therefore, a revised deep parotid lobectomy was subsequently performed. Patient provided written informed consent.

## Case report

**Figure 1:**
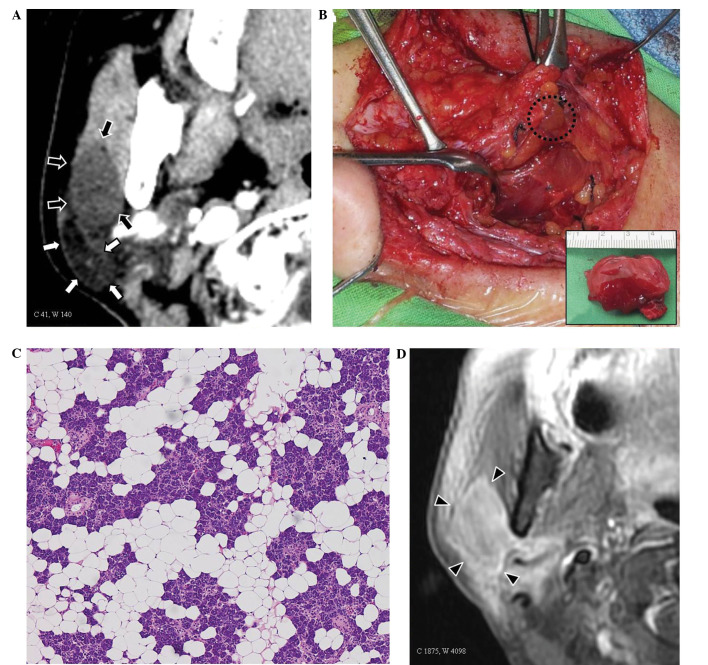
(A) Computed tomography demonstrated (closed arrows) a mass that was enhanced to a greater extent compared with (open arrows) the superficial parotid gland lobe in the right parotid region. (B) During the right superficial parotidectomy (broken circle) a soft, well-circumscribed mass was noted in the tail of the superior parotid gland lobe and was subsequently excised from the parotid gland (B, inset). (C) Histopathology demonstrated adipose cells that were assembled in a trabecular pattern amongst the glandular cells (magnification, ×100; stain, hematoxylin and eosin). (D) T1-weighted magnetic resonance imaging demonstrated (closed arrowheads) a gadolinium-enhanced mass in the right parotid region.

A 65-year-old female presented with a painless, right infra-auricular mass, which had been growing slowly over four months. Physical examination showed a 3×2-cm palpable non-tender, round, mobile and elastic mass in the patient’s right parotid gland. No other neck lesions were observed. Computed tomography (CT) demonstrated a 2.3×1.7×1.9-cm well-circumscribed and mildly enhanced lesion, located between the right parotid gland and the right masseter muscle (Fig. 1A). Therefore, a superficial parotidectomy was recommended. A 3×3×3-cm brown, soft, and well-circumscribed mass was excised en bloc (Fig. 1B). Consistent with sialolipoma, histopathological examination revealed mature adipose tissue and glandular elements that were surrounded by a fibrous capsule (Fig. 1C).

After three months, the patient experienced painful swelling in the right parotid gland. Magnetic resonance imaging (MRI) revealed a 2.5×1.7×2.6-cm cystic and ovoid lesion in the deep lobe of the right parotid space, which indicated that a postoperative sialocele had developed, which obstructs the flow of saliva (Fig. 1D). A revised deep parotid lobectomy was performed on the inner surface of the mandible ramus. A 2×2×2-cm soft and well-defined mass located in the parotid gland was removed. Histopathology of the removed mass further supported a diagnosis of sialolipoma. The subsequent six months since the surgery have been uneventful and no recurrence has been observed.

## Discussion

In the present case report, a lesion, which was later identified as a sialolipoma, was detected in the parotid gland of a 65-year-old female patient using CT. Sonography is the preferred imaging method for the majority of neck masses, however, CT and MRI may be more effective for diagnosing a parotid gland neoplasm (1,2,6). Generally, sialolipoma appear as a well-circumscribed lesion during high-resolution CT or a high-intensity MRI (1,6). In the present case, CT revealed a mass that was enhanced to a greater extent than the superficial lobe of the parotid gland in the right parotid region (Fig. 1A). The mass was irregularly shaped, fatty and contiguous with multiple soft tissue density regions. In a previous report, MRI did not differentiate the tumor capsule from the fibrous septa of cheek subcutaneous tissue in a congenital sialolipoma (3). In the present study, MRI indicated that the tumor had extended into the subcutaneous fat. It is particularly difficult to differentiate with any certainty between a benign neoplasm and a sialolipoma using CT or MRI. Therefore, a superficial parotidectomy was recommended. Superficial parotidectomy, or total parotidectomy with entire surgical removal of the tumor, is recommended for the majority of parotid gland sialolipoma. However, simple surgical excision has also been conducted in cases of minor salivary gland sialolipoma (1,3). Excision of the entire tumor is vital and a total lobectomy is required for superficial multifocal lesions, which extend into the deep lobular area (1,4,6).

Microscopically, sialolipoma exhibit a unique feature; salivary gland lesions that are entrapped within predominately extensive mature adipose tissue. This differentiates sialolipoma from adenolipoma, lipomatosis, true lipoma, fibrolipoma and spindle cell lipoma (1). The histopathogenesis of sialolipoma remains unclear (3,5); however, it has been hypothesized that sialolipoma may be associated with salivary gland dysfunction, which results in salivary gland alterations (5). According to the literature, none of the 30 cases of sialolipoma experienced recurrence following a conservative surgical excision (2–5). Thus, the present case is unique as the patient experienced recurrence despite a total excision of the tumor. The recurrence may have originated from a novel and independent sialolipoma as a residual sialolipoma was considered to be unlikely according to the pathology report, which indicated a clear safety margin.

In conclusion, postoperative follow-up is considered to be a necessity, regardless of whether a pathological examination indicates a successful excision with clear safety margins. The present case demonstrates that although surgical excision is generally sufficient to treat parotid gland sialolipoma, postoperative follow-up is required as multifocal lesions may potentially remain, which could result in recurrence.
